# Preoperative plasma D-Dimer level is correlated with peritoneal cancer index of patients with pseudomyxoma peritonei

**DOI:** 10.1186/s12893-022-01812-8

**Published:** 2022-10-31

**Authors:** Jing Feng, Changhai Qi, Yiyan Lu, Hongjiang Wei, Guowei Liang, Ruiqing Ma, Mingjian Bai

**Affiliations:** 1 Department of Clinical Laboratory, Aerospace Center Hospital, 15 Yuquan Road, Haidian District, 100049 Beijing, China; 2 Department of Pathology, Aerospace Center Hospital, 100049 Beijing, China; 3 Department of Radiology, Aerospace Center Hospital, 100049 Beijing, China; 4 Department of Myxoma, Aerospace Center Hospital, 100049 Beijing, China

**Keywords:** Tumor marker, Burden, Pseudomyxoma peritonei

## Abstract

**Purpose:**

Accurate assessment of preoperative tumor burden contribute to formulate a scientific surgical plan for patients with pseudomyxoma peritonei (PMP). Present study aimed to assess whether the preoperative plasma D-Dimer level could reflect tumor burden for PMP patients.

**Methods:**

A total of 253 PMP patients were included between June 1, 2013 and March 1, 2022. According to the peritoneal cancer index (PCI), all participants were divided into extensive (PCI ≥ 28) and none-extensive (PCI < 28) subgroups. The D-Dimer and tumor markers were compared between the two subgroups. The correlation between the abovementioned biomarkers and PCI will be calculated, and further compared with each other. Two-sided *P* value less than 0.05 is considered statistically significant.

**Results:**

The level of D-Dimer (ng/ml) between extensive and none-extensive subgroup were 600 (328, 1268) vs. 339 (128, 598), *Z* = -5.425, *p* < 0.001. The Spearman correlation between D-Dimer, carcinoembryonic antigen (CEA), carbohydrate antigen 125 (CA 125), CA 19 − 9 and PCI were 0.487, 0.509, 0.469, and 0.499, respectively (all *p* < 0.001). The correlation coefficients were compared with each other according to Meng, Rosenthal and Rubin’s method, however, there was no significant difference.

**Conclusion:**

Preoperative plasma D-Dimer could moderately reflect tumor burden for PMP. In the future, a multivariate prediction model will be developed to help surgeons to formulate a more precise surgical plan for the PMP patients.

## Introduction

Pseudomyxoma peritonei (PMP) is a rare abdominal cancer characterized by extensive growth of mucinous tumor in the peritoneal cavity[1]. The reliable epidemiological data is still difficult to determine until now, a recent survey suggested that the prevalence rate of PMP was 22 people per million per year in European countries[2]. The main characteristic feature of PMP is the abundant secretion of mucinous ascites, which slowly fills the peritoneal cavity and leads to abdominal distension[3]. Complete cytoreduction surgery (CRS) in combination with hyperthermic intraperitoneal chemotherapy (HIPEC) has been recommended as the standard treatment for PMP [4].

The peritoneal involvement extent of PMP patients was quantified by the peritoneal cancer index (PCI) [5], which is a surgical variable and commonly been used to determine the feasibility of tumor reduction [6].

Furthermore, former study reported a higher PCI was correlated with poor prognosis of PMP patients [7]. Therefore, accurate preoperative determination of the PCI is critical to optimize the selection of patients who will benefit from CRS. Unfortunately, the standard PCI calculations could only be done during surgery.

To date, several investigational modalities could reflect tumor burden of peritoneal surface malignancy and identify appropriate surgical candidates. For instance, computed tomography (CT), magnetic resonance imaging (MRI), and serum tumor markers [8]. CT was the fundamental imaging modality, the former research [9] showed a moderate correlation between the CT-PCI and surgical PCI with a correlation coefficient of 0.65, however, CT in predicting of PCI is related to the experience of the radiologist [10]. A former study had demonstrated the number of elevated tumor markers was positively correlated with preoperative PCI [11]. Further research calculated the correlation of absolute tumor marker levels with PCI in PMP patients, specifically, the correlation coefficients was 0.29 for carcinoembryonic antigen (CEA), 0.36 for carbohydrate antigen 19 ? 9 (CA 19 ? 9), and 0.42 for CA 125, nevertheless, the correlation between the three tumor markers and PCI is general [12].

We believe that multiple linear regression model might predict the preoperative PCI accurately for PMP patients, rather than relying on a single index. Consequently, more indicators which have good correlation with PCI need to be discovered before multivariate regression analysis. A former study found that the D-Dimer level was significantly higher in high PCI group than that of low PCI group in peritoneal metastasis of gastric cancer patients [13]. Thus, we speculate that D-Dimer might also correlate with PCI in PMP patients.

Present study aimed to calculate the correlation coefficients between D-Dimer and PCI, subsequently, the strength of correlation with PCI will be compared between D-Dimer and the commonly used tumor markers.

## Materials and methods

### Patients

The present study was approved by Institution Review Board (IRB) of Aerospace Center Hospital (NO. 2022-002). All data were retrieved from the follow-up database of Myxoma Department in Aerospace Center Hospital between June 1, 2013 and March 1, 2022, which was the largest single PMP center in China. PMP diagnosis was confirmed by two experienced pathologists according to the Peritoneal Surface Oncology Group International (PSOGI) criteria [14], as the criteria was published in 2016, pathologist in our center reviewed all the pathology specimens before 2016.

**Fig. 1:**
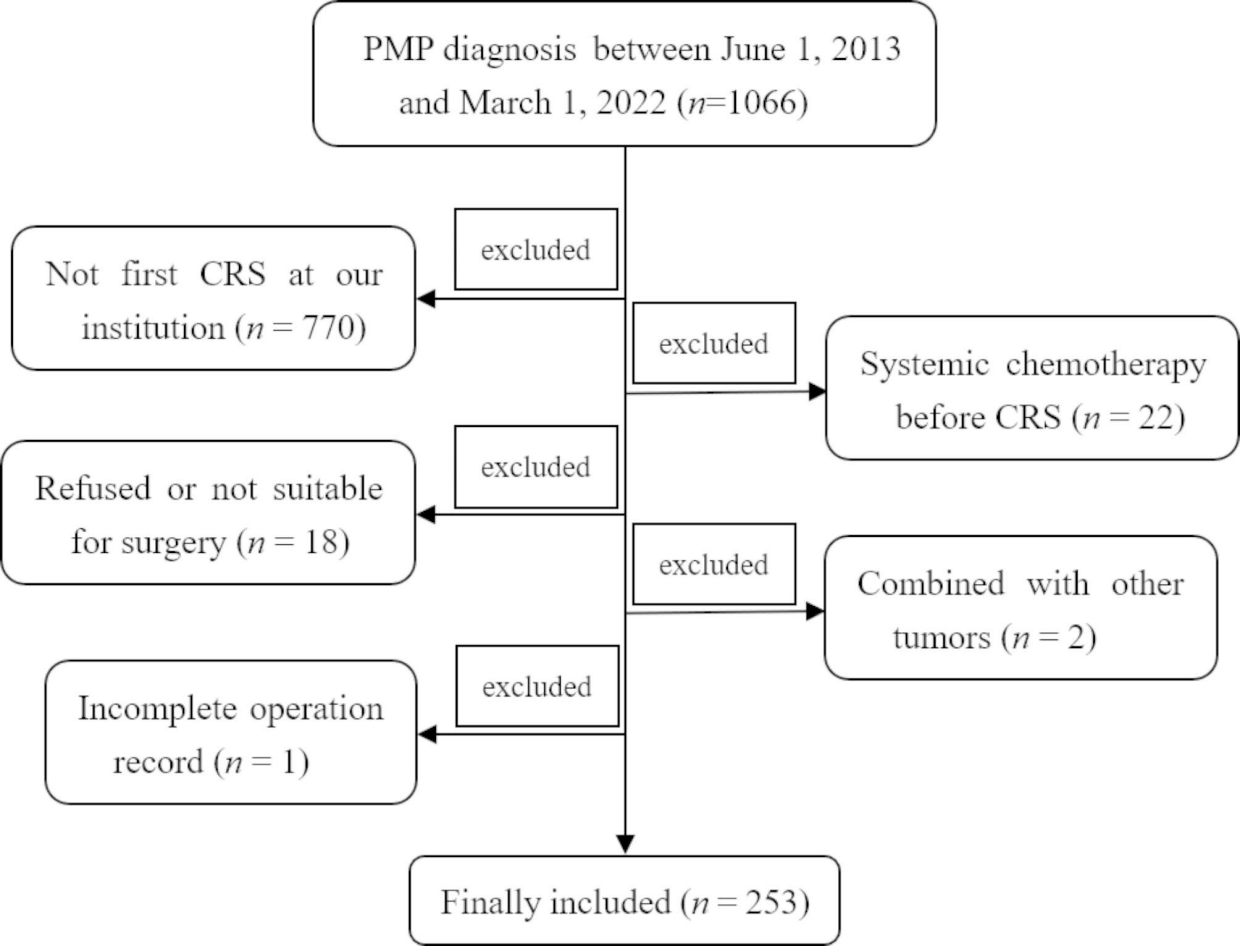
Study schematic. A total of 1066 PMP patients were retrieved in the follow-up database. 770 patients not underwent CRS in our institution for first time were excluded. 22 patients received systemic chemotherapy before CRS, 18 patients refused or not suitable for CRS, 2 patients combined with other tumors, and one with incomplete operation record were also excluded. Ultimately, 253 patients were included PMP: pseudomyxoma peritonei; CRS: cytoreductive surgery

A total of 1066 subjects with PMP diagnosis were retrieved from our database. The exclusion and inclusion criteria were as follows. The exclusion criteria including: (a) Patients whose first time CRS not performed in our center (*n* = 770); (b) Patients received systemic chemotherapy before CRS (*n* = 22); (c) Although PMP diagnosis was confirmed by puncture biopsy, the patient refused further surgery or the doctor thought the patient was not suitable for surgery (*n* = 18); (d) Patients also suffered from other types of tumors (*n* = 2, one patient with breast cancer, while another one with both breast and thyroid cancer); (e) Patients with incomplete operation record (*n* = 1). Patients whose CRS were performed in our center for the first time was considered as the inclusion criteria. Ultimately, 253 participants were included (Fig. 1). According to the PCI level, all PMP patients were divided into extensive PMP subgroup (PCI ≥ 28) and non- extensive PMP subgroup (PCI < 28), respectively [15].

### Biomarker determination

All biomarkers were tested before CRS and performed according to manufacturer’s instructions. Plasma D-Dimer was determined by Immunoturbidimetry (ACL TOP 700, America) and serum tumor markers (including CEA, CA 125, and CA 19 − 9) were tested by chemiluminescence immunoassay (CMIA) method (Abbott, America). All biomarkers underwent Internal Quality Control (IQC) and External Quality Assessment (EQC), in order to ensure the accuracy of results.

### PCI calculation

PCI score calculation was performed by comprehensive abdominal exploration according to Sugarbaker’s criteria [16]. The PCI scoring system divides the abdomen into thirteen areas, a score of 0–3 is given for each of the 13 areas (0 for no tumor, 1 for nodules < 0.5 cm, 2 for nodules between 0.5 and 5 cm, and 3 for nodules > 5 cm). The total score is then calculated by adding all the scores, with ranges from 0 to 39.

### Statistical analysis

Statistics analyses were performed by *SPSS* (version 16.0; IBM Corporation, Armonk, NY, USA) and *R* (version 4.0.2; R Foundation for Statistical Computing, Vienna, Austria). *Chi-square* test was used to compare rate differences between groups, while continuous data between groups was compared by independent *T* test or *Mann-Whitney U* test, as appropriate.

All the participated biomarkers levels were compared between extensive and non-extensive PMP subgroup, respectively. *Pearson* or *Spearman* correlation coefficients were calculated between biomarker levels and PCI depending on their normality, subsequently, the correlation heat map were plotted by *R*. Correlation coefficient values > 0.7 be regarded as “strong” correlation, values between 0.50 and 0.70 be interpreted as “good” correlation, between 0.3 and 0.5 be treated as “moderate” correlation, and any value < 0.30 would be poor correlation [17]. Subsequently, statistical comparison of correlations was performed according to Meng, Rosenthal, and Rubin’s method [18], by which, the better biomarkers will be suggested for predicting tumor burden in PMP patients. Two-sided *P* value less than 0.05 is considered statistically significant difference.

## Results

A total of 253 patients were included in present study. There were 145 males and 108 females, the mean age was 58 ± 11 years. The *median (IQR)* duration from biomarkers detection to CRS was 7 (5, 10) days. The *median* PCI was 27 (17, 33). There were 94 participants underwent complete cytoreduction, while 159 underwent debulking surgery (Table 1). There were 119 patients in extensive PMP (9 underwent complete cytoreduction and 110 underwent debulking surgery) and 134 patients in non-extensive PMP subgroup (85 underwent complete cytoreduction and 49 underwent debulking surgery), χ^2^ = 100.7, *P* < 0.001, respectively.

**Table 1:** Baseline characteristics of 253 included PMP patients

Clinicopathological variables	
Sex (Male/Female)	145/108
Age (years)	58 ± 11
Operation time (hours)	7.7 ± 2.2
Operation method	
Complete cytoreduction (*n*)	94
Debulking surgery (*n*)	159
Hospital time (days)	24.8 ± 7.9
PCI	27 (17, 33)
Histological subtype	
Acellular mucin (*n*)	1
DPAM (*n*)	194
PMCA (*n*)	39
PMCA-S (*n*)	19
D-Dimer (collected/missed)	252/1
CEA (collected/missed)	253/0
CA 125 (collected/missed)	253/0
CA 19 − 9 (collected/missed)	253/0

*PMP* pseudomyxoma peritonei, *PCI* peritoneal cancer index, *DPAM* diffuse peritoneal mucinous adenoma, *PMCA* peritoneal mucinous carcinoma, *PMCA-S* peritoneal mucinous carcinomatosis with signetring cells

**Fig. 2:**
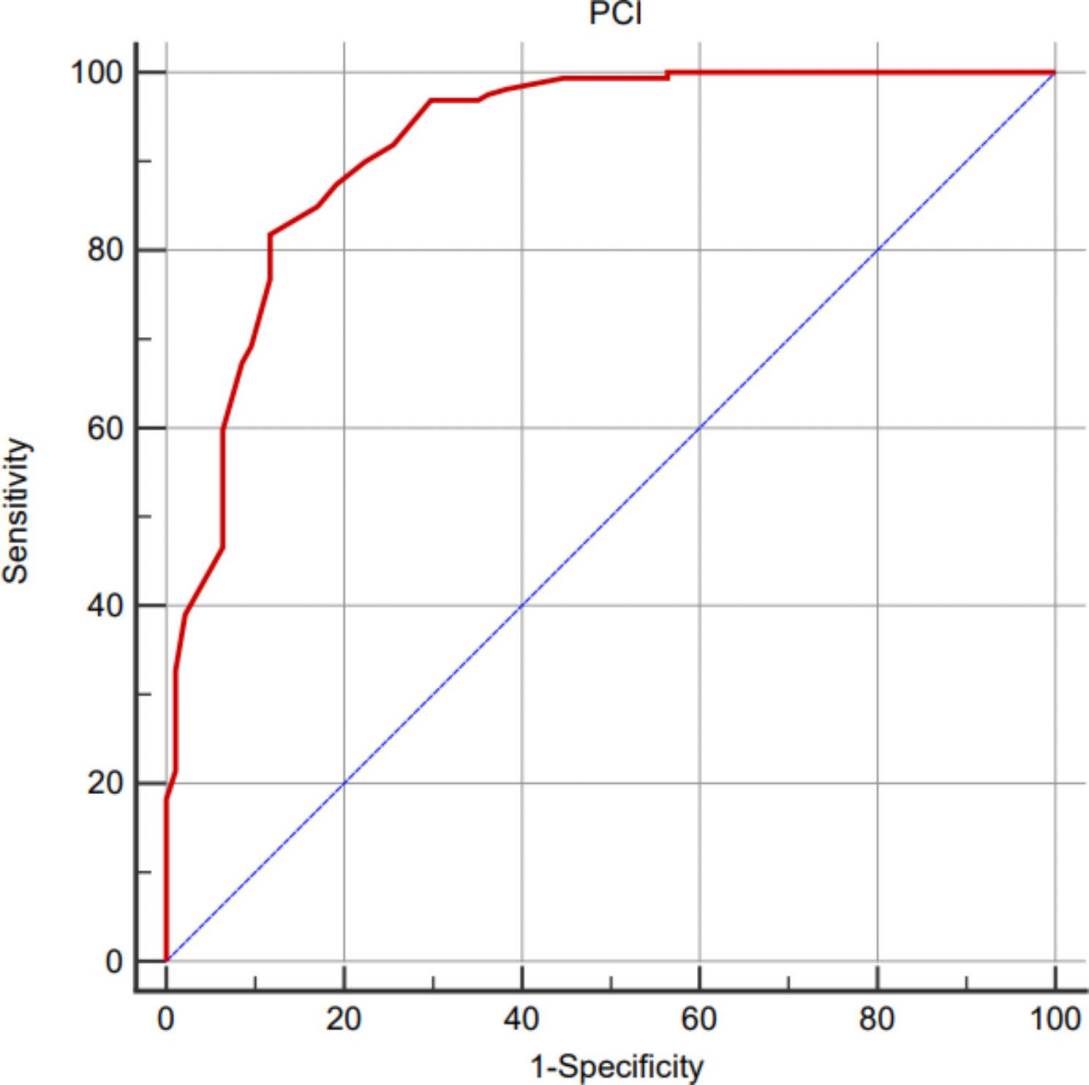
Optimal cut-off value of PCI to predict complete cytoreduction for PMP patients

All biomarkers and PCI were tested for normality, unfortunately, none of them corresponding to normal distribution. The level of D-Dimer (ng/ml), CEA (ng/ml), CA 125 (U/ml), and CA 19 − 9 (U/ml) between extensive and none-extensive group were[600 (328, 1268) vs. 339 (128, 598), *Z* = -5.425, *p* < 0.001], [27.50 (12.43, 95.39) vs. 6.58 (1.75, 44.92), *Z* = -5.553, *p* < 0.001], [75.30 (48.40, 162.32) vs. 38.52 (11.92, 91.20), *Z* = -5.271, *p* < 0.001], and [102.10 (21.93, 350.57) vs. 14.55 (5.23, 67.78), *Z* = -6.058, *p* < 0.001], respectively (Table 2). The optimal cut-off value of PCI to predict complete cytoreduction was 25, with the AUC-ROC of 0.919 (95%*CI*: 0.878 ∼ 0.949) (Fig. 2).

**Fig. 3:**
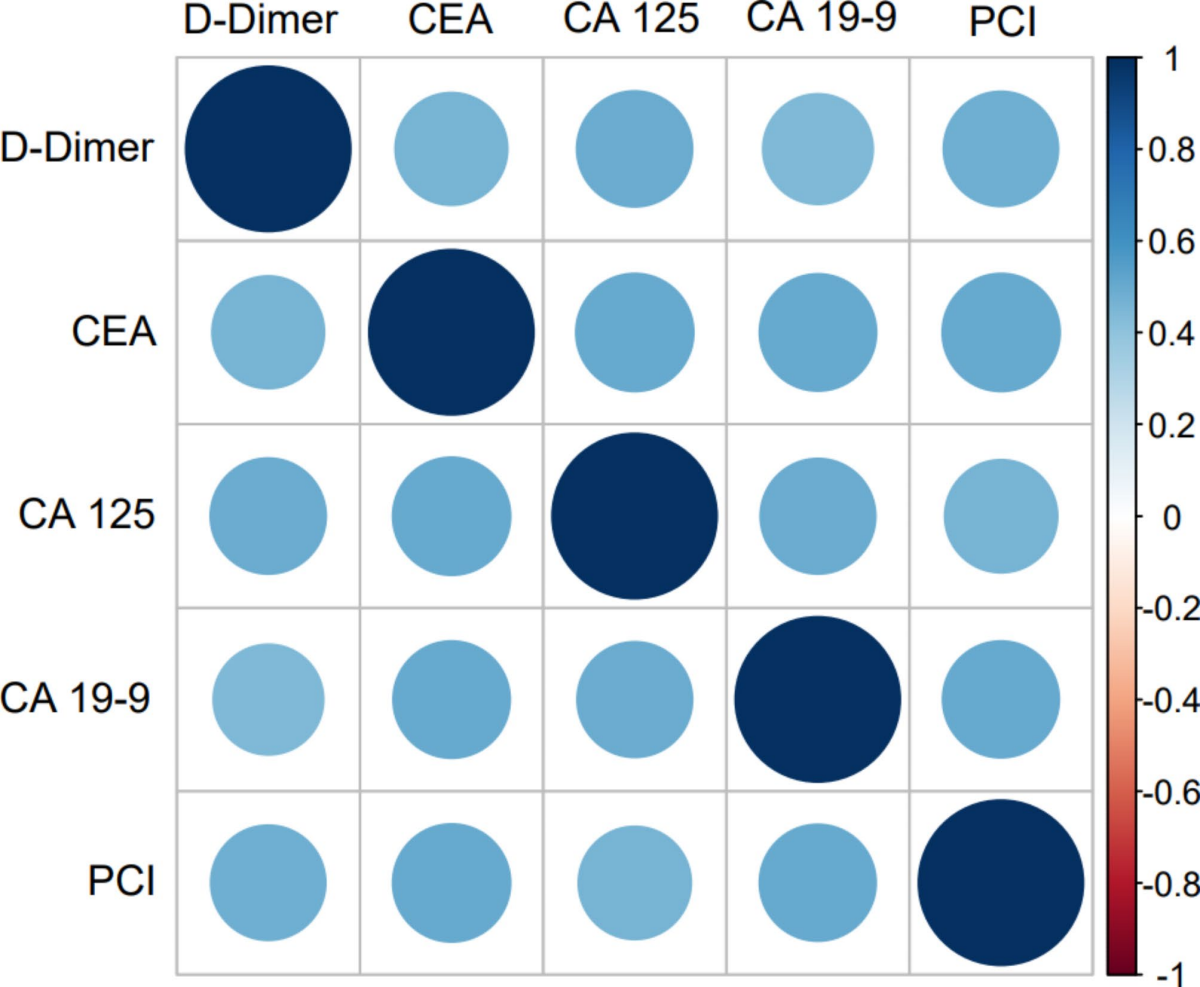
Correlation heat map among D-Dimer, tumor markers and PCI.

The *Spearman* rank correlation between D-Dimer, CEA, CA 125, CA 19 − 9 and PCI were 0.487, 0.509, 0.469, and 0.499 (all *p* < 0.001), respectively (Table 3 and the Fig. 3). Subsequently, the correlation coefficients were compared with each other according to Meng, Rosenthal and Rubin’s method, however, there was no significant difference in the size of correlation coefficient, details were shown in Table 4.

**Table 2:** Biomarkers levels between extensive and none-extensive PMP group

Biomarkers	Extensive PMP (*n* = 119)	None-extensive PMP (*n* = 134)	*Z*	*P*-value
D-Dimer (ng/ml)	600 (328, 1268)	339 (128, 598)	-5.425	0.001
CEA (ng/ml)	27.50 (12.43, 95.39)	6.58 (1.75, 44.92)	-5.553	0.001
CA 125 (U/ml)	75.30 (48.40, 162.32)	38.52 (11.92, 91.20)	-5.271	0.001
CA 19 − 9 (U/ml)	102.10 (21.93, 350.57)	14.55 (5.23, 67.78)	-6.058	0.001

*PMP* pseudomyxoma peritonei

**Table 3:** *Spearman* rank correlation among biomarkers and PCI in PMP patients (all *P* < 0.001)

	D-Dimer	CEA	CA 125	CA 19 − 9	PCI
D-Dimer	1.000	0.463	0.492	0.445	0.487
CEA	0.463	1.000	0.509	0.503	0.509
CA 125	0.492	0.509	1.000	0.490	0.469
CA 19 − 9	0.445	0.503	0.490	1.000	0.499
PCI	0.487	0.509	0.469	0.499	1.000

*PCI* peritoneal cancer index, *PMP* pseudomyxoma peritonei

**Table 4:** Statistical comparison of correlations between biomarkers and PCI in PMP patients (all *P* > 0.05)

	D-Dimer	CEA	CA 125	CA 19 − 9
D-Dimer	─			
CEA	*z* = 0.406 (*p* = 0.685)	─		
CA 125	*z* = 0.334 (*p* = 0.738)	*z* = 0.762 (*p* = 0.446)	─	
CA 19 − 9	*z* = 0.217 (*p* = 0.828)	*z* = 0.192 (*p* = 0.848)	*z* = -0.559 (*p* = 0.576)	─

*PCI* peritoneal cancer index, *PMP* pseudomyxoma peritonei

## Discussion

Present study found that plasma D-Dimer was significantly higher in extensive PMP subgroup than that of none-extensive PMP subgroup. There was a moderate correlation between D-Dimer level and PCI, however, compared with commonly used three tumor markers, the correlation coefficient size did not reach statistical significance.

The PCI is always performed during surgery for PMP patients [19]. Therefore, accurate preoperative assessment of disease burden is essential to avoid nontherapeutic laparotomies in many patients or withholding of potentially beneficial therapy in others. The application value of D-Dimer in tumor has been confirmed by extensive researches [20, 21]. In 2018, a meta-analysis included 13,001 patients, which found an elevated D-Dimer was markedly associated with poor overall survival (OS) and shorter progression-free survival (PFS) [22]. We also discovered that the elevated D-Dimer was the independent prognostic risk factor for PMP patients [23].

By searching the literature, present study first evaluated the correlation between D-Dimer and PCI, however, the correlation was moderate. Therefore, we speculate that D-Dimer, like other tumor markers, can moderately reflect the tumor load for PMP patients before operation. In future research, we should pay attention to the level of D-Dimer in PMP.

All the three tumor markers had a moderate correlation with PCI, this result is similar with the previous study [12]. After statistical comparison of coefficients, D-Dimer did not show statistical significance with the other three tumor markers, which indicating that there was no difference between D-Dimer and tumor markers in reflecting tumor burden for PMP patients. Therefore, it seems that it is not appropriate to predict PCI by a single index. We believe that a predictive model should be developed to predict PCI preoperatively for PMP patients in the future, with the predictors including clinical features, D-Dimer, tumor markers, and preoperative CT. The established prediction model might be more accurately predict the PCI level and help surgeons formulate a more elaborate surgical plan before operation.

There were two limitations in present study. First, the majority of PMP patients whose first time CRS were not performed in our center, in order to ensure the quality of the study, these patients were excluded from our research, consequently, which might lead to selection bias. Second, due to the defects of retrospective study, there were many missing data of CA 724 and CA 242, which were not assessed in the present study.

## Conclusion

To conclude, the plasma D-Dimer could moderately reflect tumor burden, which is similar to CEA, CA 125, and CA 19 − 9. However, there should be a strong correlation (> 0.70) to use for any clinical application. In the future, a prediction model including these predictors will be developed, which may help surgeons to develop a more precise surgical plan for the PMP patients.

## Data Availability

All data generated or used during the study are available from the corresponding author by request.
